# Factors Influencing COVID-19 Vaccine Hesitancy among Patients with Serious Chronic Illnesses during the Initial Australian Vaccine Rollout: A Multi-Centre Qualitative Analysis Using the Health Belief Model

**DOI:** 10.3390/vaccines11020239

**Published:** 2023-01-20

**Authors:** Tammie Choi, Bryan Chan, Lisa Grech, Alastair Kwok, Kate Webber, Jennifer Wong, Mike Nguyen, Nathan Bain, Daphne Day, Amelia McCartney, Ruby Hamer, Eva Segelov, on behalf of the CANVACCS DIABVACCS and MSVACCS Investigators

**Affiliations:** 1 Department of Nutrition, Dietetics, and Food, School of Clinical Sciences, Monash University, Notting Hill, VIC 3168, Australia; 2 Department of Oncology, Sunshine Coast Hospital and Health Service, Birtinya, QLD 4575, Australia; 3 School of Medicine and Dentistry, Griffith University, Birtinya, QLD 4575, Australia; 4 Department of Medicine, School of Clinical Sciences, Monash University, Clayton, VIC 3168, Australia; 5 Department of Oncology, Monash Health, Clayton, VIC 3168, Australia; 6 Department of Diabetes and Vascular Medicine, Monash Health, Clayton, VIC 3168, Australia; 7 Department of Clinical Research, Faculty of Medicine, University of Bern, 3012 Bern, Switzerland

**Keywords:** COVID-19, vaccine hesitancy, qualitative analysis, health belief model, chronic illness, cancer, diabetes, multiple sclerosis

## Abstract

**Background**: People with chronic illnesses have increased morbidity and mortality associated with COVID-19 infection. The influence of a person’s serious and/or comorbid chronic illness on COVID-19 vaccine uptake is not well understood. **Aim**: To undertake an in-depth exploration of factors influencing COVID-19 vaccine uptake among those with various serious and/or chronic diseases in the Australian context, using secondary data analysis of a survey study. **Methods**: Adults with cancer, diabetes and multiple sclerosis (MS) were recruited from 10 Australian health services to undertake a cross-sectional online survey (30 June to 5 October 2021) about COVID-19 vaccine uptake, vaccine hesitancy, confidence and complacency and disease-related decision-making impact. Free-text responses were invited regarding thoughts and feelings about the interaction between the participant’s disease, COVID-19, and vaccination. Qualitative thematic analysis was undertaken using an iterative process and representative verbatim quotes were chosen to illustrate the themes. **Results**: Of 4683 survey responses (cancer 3560, diabetes 842, and MS 281), 1604 (34.3%) included free-text comments for qualitative analysis. Participants who provided these were significantly less likely to have received a COVID-19 vaccination than those who did not comment (72.4% and 86.2%, respectively). People with diabetes were significantly less likely to provide free-text comments than those with cancer or MS (29.0%, 35.1% and 39.9%, respectively). Four key themes were identified from qualitative analysis, which were similar across disease states: (1) having a chronic disease heightened perceived susceptibility to and perceived severity of COVID-19; (2) perceived impact of vaccination on chronic disease management and disease-related safety; (3) uncertain benefits of COVID-19 vaccine; and (4) overwhelming information overload disempowering patients. **Conclusions**: This qualitative analysis highlights an additional layer of complexity related to COVID-19 vaccination decision making in people with underlying health conditions. Appreciation of higher susceptibility to severe COVID-19 outcomes appears to be weighed against uncertain impacts of the vaccine on the progression and management of the comorbid disease. Interactions by clinicians addressing individual factors may alleviate concerns and maximise vaccine uptake in people with significant underlying health conditions.

## 1. Introduction

Immunisation remains the cornerstone of population health measures against preventable illnesses; however, effectiveness relies on large scale acceptance of the vaccine [1,2]. With the emergence of the severe acute respiratory syndrome coronavirus 2 (SARS-CoV-2) global pandemic, effective vaccines were produced at a record pace [2,3].

The issue of vaccination is complex for people with underlying medical conditions who need to balance their heightened vulnerability from novel coronavirus disease 2019 (COVID-19) infection with concerns about the impact vaccines may have on their treatment or disease course [4,5,6]. Early in the pandemic, reports confirmed that those with underlying conditions were more vulnerable to infection and more likely to experience severe complications [7]. As a result, this group was prioritised to receive the vaccine in many countries, including Australia, where just under half of the population (11.6 million) lives with at least one chronic condition [8].

Vaccine hesitancy is defined as a “delay in acceptance or refusal of vaccination despite availability of vaccination services” [9]. Hesitancy develops for many reasons, including concern about side effects [10,11], perceived lack of safety [12], and from dissemination of misinformation [13], with reasons varying according to context-specific factors [14].

A detailed understanding of factors influencing hesitancy in people with underlying health conditions is critical. The rapid evolving nature of the COVID-19 pandemic led to high levels of uncertainty, stress, and anxiety, as communities were required to change current and future behavior [15]. People living with chronic illness during the rapidly evolving pandemic reported substantial negative mental health impacts, including feelings of fear and anxiety related to vulnerability to COVID-19 infection and complications and social isolation associated with minimising social contact to protect themselves from COVID-19 [16]. To support our understanding of vaccine hesitancy gained from quantitative research [17,18], in-depth qualitative analysis is important for a nuanced understanding about how to best support some of the most medically vulnerable populations. Indeed, the aim of qualitative research is to understand beliefs, experiences, attitudes and behaviors, as well as how these interact [19]. There is limited qualitative research exploring the factors that influence COVID-19 vaccine hesitancy in people with different chronic diseases [20]. In particular, qualitative studies that have used a theoretical framework to inform strategies for addressing COVID-19 vaccine hesitancy in this population are lacking. The Health Belief Model (HBM) has been widely used as a theoretical construct to help understand health behaviour [21]. It describes the influence of personal knowledge, beliefs, and perceptions about a disease and the strategies to avoid it. Behavioural change is only expected if an individual perceives that the benefits outweigh risks. The HBM can be adapted to better understand, and thus specifically address, the multiple factors influencing an individual’s willingness or hesitancy to receive vaccines [22,23,24].

We conducted a large, multi-centre Australian survey study of people with cancer, diabetes, and multiple sclerosis (MS) to evaluate attitudes towards uptake and hesitancy at the time that COVID-19 vaccines first became available. The aims of this secondary data analysis were to perform qualitative analysis of free-text comments from this survey study to: (1) explore the factors that underpin COVID-19 vaccine hesitancy in people with chronic illnesses; and (2) assess these factors influencing COVID-19 behaviour using the Health Belief Model.

## 2. Materials and Methods

### 2.1. Participants and Procedures

Data were collected as part of a larger study survey that has been described in detail elsewhere, including results of the quantitative analysis from survey scales [4]. Here, we present the thematic analysis of participants’ free-text responses from participants with one of three conditions: cancer, diabetes or MS. Briefly, participants were invited to complete an online survey assessing COVID-19 vaccine uptake, hesitancy, and beliefs focusing on the context of their comorbidities and how this influenced their complacency and concerns.

**Figure 1:**
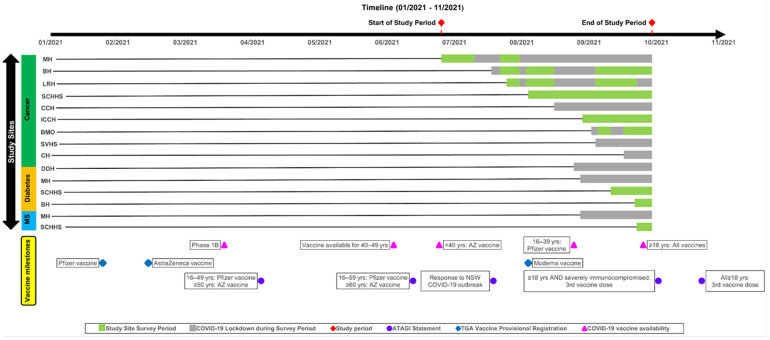
Timeline of study including survey period relative to statewide strict lockdowns for COVID-19 and vaccine milestones. Abbreviations: MH, Monash Health; BH, Bendigo Health; LRH, Latrobe Regional Hospital; SCHHS, Sunshine Coast Hospital and Health Service; CCH, Central Coast Hematology; ICCH, Icon Cancer Centre Hobart; BMO, Border Medical Oncology; SVHS, St Vincent’s Hospital Sydney; CH, Campbelltown Hospital; DDH, Dr David Hoffman; Yrs, Years; AZ, Astra-Zeneca; NSW, New South Wales; ATAGI, Australian Technical Advisory Group on Immunization; TGA, Therapeutic Goods Administration—COVID-19 Vaccine Provisional Registration. People diagnosed with either cancer, diabetes, or MS were eligible for COVID-19 vaccination from the commencement of the Australian Government Rollout Phase 1B.

Recruitment of people aged over 18 years of age and older with a diagnosis of either cancer, diabetes, or MS was via text message or direct invitation across 10 health services (five regional/rural) in four states in Australia between 30 June and 5 October 2021 (Figure 1). People with cancer and diabetes were invited if they were scheduled to attend an appointment in the upcoming six months, whereas people with MS were invited if they attended an appointment in the previous 12 months. The text message invitation contained a link to the Participant Information and Consent Form and the survey. People with cancer were also invited at their consultations and treatment appointments, and from advertisement materials displayed at the health services. The survey was presented in English but assistance for non-English speakers from family and friends was encouraged. Data capture was hosted by Qualtrics^®^ secure platform (SAP, New York, NY, USA). Paper surveys were provided upon request.

### 2.2. Measures

#### 2.2.1. Quantitative Measures

The following were collected: gender, age, highest level of education, annual household income, English as dominant language, geographical location, diagnosis, disease subtype and/or stage, time since diagnosis, disease status, and current treatment. COVID-19 vaccination status including number of doses was self-reported. The survey collected demographic and disease-related information and responses to three validated scales: the Oxford COVID-19 Vaccine Hesitancy Scale; the Oxford COVID-19 Vaccine Confidence and Complacency Scale; and the Disease Influenced Vaccine Acceptance Scale-Six [25,26].

#### 2.2.2. Qualitative Responses

Following survey responses, respondents were invited to “Please include any comments about your feelings and thoughts about your [cancer; diabetes; or MS] and COVID-19 vaccination that you would like to share. If you have no comments to include, please type ‘Nil’”.

### 2.3. Data Analysis and Interpretative Framework

Data cleaning was performed to remove incomplete survey responses and correct spelling errors within the free-text comments. Descriptive analysis was undertaken to describe characteristics of participants who provided qualitative comments. Between-group analysis using chi-squared tests for independence was undertaken to identify differences between participants who did and did not comment. Statistical analyses were performed using SPSS Version 27.0 (IBM, Armonk, NY, USA).

#### 2.3.1. Qualitative Analysis

Free-text survey responses were qualitatively analysed if participants provided comments (“Nil” or “No Comments” were excluded). Qualitative content analysis was undertaken to identify key themes from free-text survey responses using computer-aided content analysis software package, Leximancer™ Version 4.5 (Leximancer, St. Lucia, Australia) [27]. This utilises machine learning algorithms to analyse large volumes of natural language and identify meanings within passages of text [28]. The software determines the main concepts within texts using word frequency statistics and co-occurrences and generates thematic visualisations as concept maps, diagrammatically linking one cluster of terms to the next [29]. In this study, the main concepts were independently evaluated for triangulated consensus on qualitative themes by three authors (TC, BC, and AK). Leximancer concept maps were explored to understand differences between participants according to their comorbid disease.

#### 2.3.2. Trustworthiness and Rigour

To enhance research rigour, investigator triangulation was applied. As Leximancer™ does not consider meaning behind the cluster of terms, the first 100 text extracts for the four highest frequency concepts were manually thematically analysed by three authors (TC, BC, and AK) before coming together to discuss their interpretation of the findings. The thematic analysis utilised authors’ individual knowledge to interpret the data. The analysis was conducted by TC—a diabetes dietitian, BC—an experienced oncologist, and AK—an oncology research officer. Furthermore, the themes and interpretation were reviewed by the authorship team who are experts in different fields, including LG—a senior research fellow specializing in MS research, JW—an endocrinologist, KW and ES—both experienced medical oncologists. Their backgrounds as healthcare providers and their clinical experience of chronic disease management influenced their interpretation and the meaning derived from the survey comments.

#### 2.3.3. Theoretical Framework

**Figure 2:**
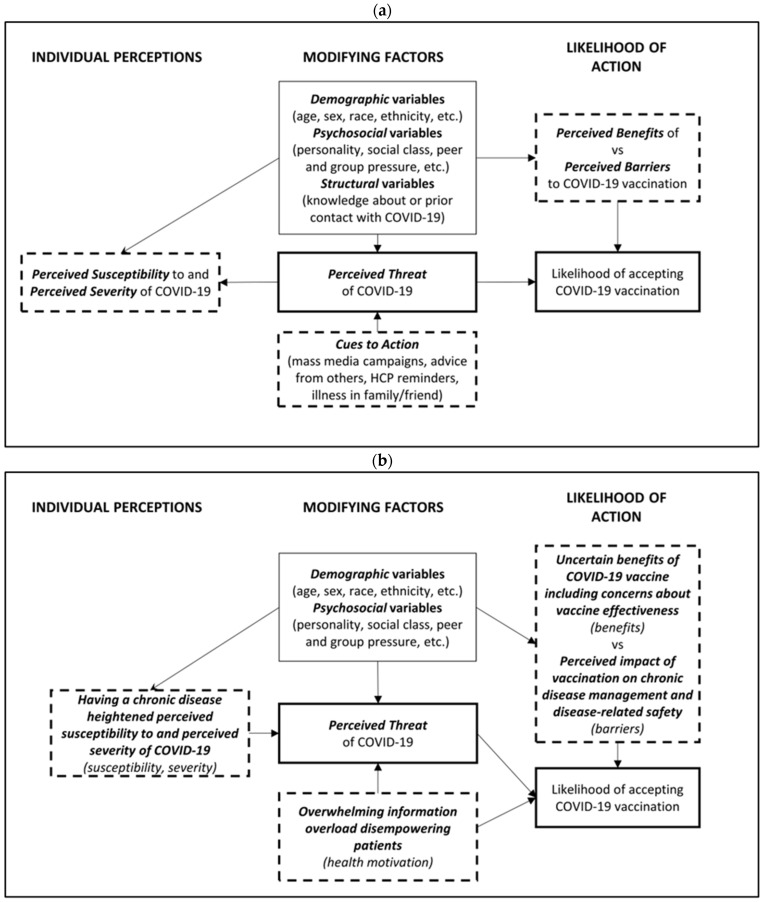
The Health Belief Model [21]; (**a**) adapted for the likelihood of COVID-19 vaccination and (**b**) adapted for the likelihood of COVID-19 vaccination with the four key themes from the qualitative analysis of free-text comments.

The HBM (Figure 2a) was used to explore the interplay between individual perceptions and modifying factors that ultimately influenced the likelihood of behavioural decision making with the qualitative analysis.

### 2.4. Consent and Ethics Statement

This research collected anonymous participant data following the provision of informed consent. The study was approved by the Monash Health Human Research Ethics Committee (RES-21-0000-364L–76466) and was registered with the Australian New Zealand Clinical Trials Registry (ACTRN12621001467820).

## 3. Results

### 3.1. Participant Characteristics

Of the 8232 survey responses, 4683 were used in analysis (3037 were submitted incomplete, 47 were duplicates, and 465 were ineligible). People with cancer made up the majority of respondents (n = 3560), followed by diabetes (n = 842), and MS (n = 281).

One thousand six hundred and four respondents (34.3% of all survey respondents) provided free-text comments able to be qualitatively analysed. Most participants who provided comments (1162 of 1604, 72.4%) self-reported having had at least one COVID-19 vaccine dose. Of the 442 unvaccinated respondents (27.6%), 45.2% stated they were likely to accept a vaccine in the future, whilst 28.7% were unlikely to do so and 26.1% were undecided. Demographic characteristics are presented in Table 1.

#### 3.1.1. Commenters vs. Non-Commenters

Free-text comments were provided by 35.1% of participants with cancer (n = 1248), 29.0% of participants with diabetes (n = 244), and 39.9% of participants with MS (n = 112). People with diabetes were less likely to provide free-text comments than those with cancer or MS (29.0%, 35.1%, and 39.9%, respectively), (*X*^2^ [2] = 15.3, *p* < 0.001). Those who provided comments were significantly less likely to have received a COVID-19 vaccination and/or to express intent to be vaccinated compared with those who did not comment (72.4% and 86.2%, respectively); this was consistent across all three co-morbidities (Tables S2–S4). Females were significantly more likely to comment than males in people with cancer (37.3% and 32.2%, respectively) and people with diabetes (32.8% and 26.3%, respectively). Free-text commenters were otherwise broadly representative of the entire survey cohort with respect to clinical and demographic characteristics (Tables S2–S4).

#### 3.1.2. Characteristics of Commenters

The clinical characteristics of those who provided free-text comments are summarised in Table S1. Around one-third came from regional centres (33.2%). As expected, due to disease biology, the MS cohort had a higher proportion of females and a lower median age compared to the cancer and diabetes cohorts. The proportion who reported English as their nondominant language varied between people with diabetes (16.8%), cancer (7.9%), and MS (10.7%).

### 3.2. Key Themes

**Figure 3:**
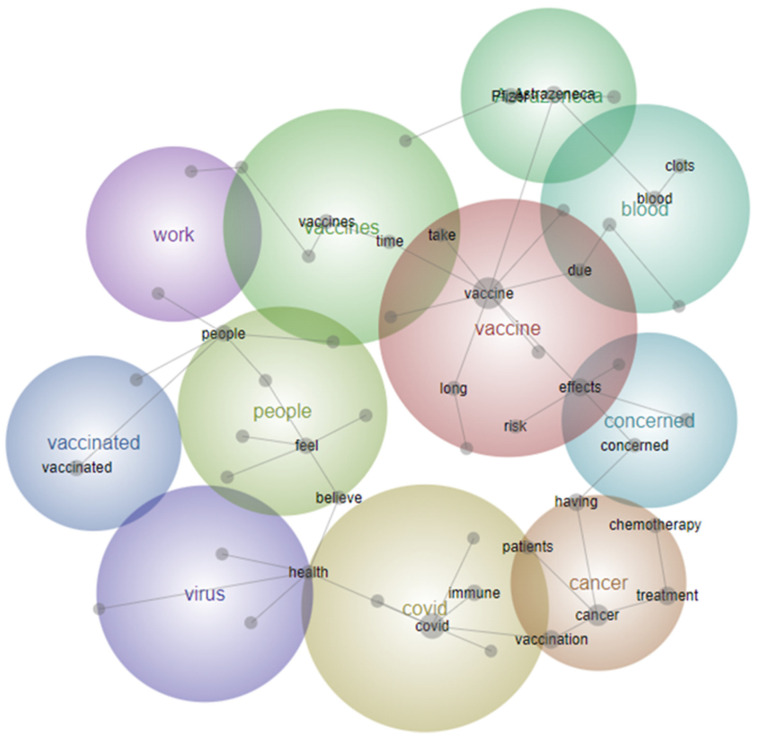
Leximancer-generated concept map of comments from people with cancer, multiple sclerosis, and diabetes.

For the qualitative analysis of free-text comments, this was a combined analysis that included a significant proportion of people with cancer. The top three concepts generated by Leximancer software (vaccine, cancer, and COVID) were manually explored to identify themes. These analyses were used to create concept maps for each disease subgroup separately (cancer, MS, and diabetes), as well as for all three combined (Figure 3). The visual maps of keywords were similar across each of the three conditions with most clusters centred around vaccine safety (particularly blood clots for patients with cancer), immunity and vaccine timing, impact on their condition, interactions between vaccine and their treatment, as well as information in general. The HBM framework was used to help organise responses into themes. Overall, four dominant themes emerged correlating with our adapted HBM (Figure 2b). Representative verbatim quotations are listed below the identified themes.

#### 3.2.1. THEME 1. Having a Chronic Disease Heightened Perceived Susceptibility to, and Perceived Severity of COVID-19

Many participants reported concerns about vulnerability due to their underlying conditions. This related both to the chance of acquiring COVID-19 due to compromised immunity and experiencing potentially more severe COVID-19 complications due to their chronic diseases. As a result, many felt that it was appropriate that they were prioritised by health authorities for COVID-19 vaccination.

*Cancer patients like myself should be given an opportunity as soon as possible whilst doing chemotherapy, as we are more vulnerable due to our weakened immune [system] and health.*

(Breast cancer, localised, female, age 47, unvaccinated).

*Got the COVID-19 shot as my immune system is weak due to type 2 diabetes.*

(Type 2 diabetes, female, age 64, vaccinated).

#### 3.2.2. THEME 2. Perceived Impact of Vaccination on Chronic Disease Management and Disease-Related Safety

The added complexity of their chronic condition, particularly for those whose disease was not well controlled, generated concerns about how a COVID-19 vaccination may complicate their chronic disease management, thus contributing to vaccine hesitancy.

*Having diabetes that isn’t well controlled at the moment, and knowledge of some of the side-effects being those that I am already [at] high risk for concerns me, so as much as I want the vaccine, I am scared to do it.*

(Type 1 diabetes, female, age 36, unvaccinated).

*Already having a weak immune system prone to infection, I obviously am scared of what COVID-19 could do, but also scared of the minimal research on short term and long-term effects of the vaccine and what that could mean for my body.*

(MS, relapsing-remitting, female, age 32, unvaccinated).

Choosing between vaccination and ‘rocking the boat’ in their chronic disease management was described extensively as a barrier to vaccine uptake.

*I had concerns about getting COVID-19 and how it would affect my MS, how sick I would get if I got it. But I also had reservations at first about having vaccination as to how my body would react and how it would affect my MS.*

(MS, relapsing-remitting, female, age 53, vaccinated).

*My MS is well controlled at this stage. So I would like to know whether [the] COVID-19 vaccine will push any of the symptoms of my MS and will make my life miserable.*

(MS, relapsing-remitting, female, age 32, unvaccinated).

*I do believe that vaccines are necessary but I’m between a rock and a hard place. I don’t know how my body will react, both with heart disease, blood clotting in my legs and diabetes.*

(Type 2 diabetes, male, age 72, unvaccinated).

*My only reservation was whether COVID-19 vaccine side effects may delay treatment. Thus [I] waited for a break in treatment before going ahead.*

(Gastrointestinal cancer, metastatic, male, age 58, vaccinated).

Many participants expressed concerns regarding the apparent ‘rushed’ development of the COVID-19 vaccine. They reported a lack of available information about disease-specific side effects, impact on existing chronic disease treatment, and long-term disease trajectory.

*So hesitancy about the vaccine is not only about its effectiveness but also about longer term impacts on MS progression. There [are] simply no studies with data available to assist with the decision.*

(MS, primary progressive, female, age 42, vaccinated).

*There [have] been no studies regarding targeted immune therapies and the COVID-19 vaccine. [As] cancer patients, our immune system is precious and this is a big unknown.*

(Lung cancer, metastatic, female, age 57, unvaccinated).

*I am concerned about recent overseas studies which have shown decreased immune response after mRNA vaccination in patients on certain cancer drugs, and in particular Imatinib.*

(Blood cancer, female, age 50, vaccinated).

*I am reluctant to take the vaccine until I have more data of the longer-term effects it has on someone with diabetes. Until then, I do not wish to risk any further inconvenience to being a diabetic.*

(Type 1 diabetes, male, age 25, unvaccinated).

These disease-specific vaccine safety concerns hindered participant self-efficacy and individual motivation to get vaccinated. Some participants reported their refusal to get vaccinated, whereas others reported feeling helpless when trying to make an informed decision about whether to accept a COVID-19 vaccine. Delaying vaccination until more information became available was reported by some participants who struggled to align their treatment or management regimens without medical advice.

*I personally have had enough with the cancer treatment and am not willing to also be a guinea pig for this vaccine.*

(Head and neck cancer, localised, male, age 60, unvaccinated).

*I honestly do not know what to do regarding vaccination. I am concerned about long term side-effects with COVID-19 vaccine.*

(Type 1 diabetes, female, age 46, unvaccinated).

For some participants with metastatic or incurable cancers, it was difficult considering a vaccine with uncertain risks and potentially limited benefit given their prognosis.

*I have [stage] 4 metastatic cancer. My immune system is shattered and I am terrified that the actual vaccine will either kill me or have a detrimental effect on my cancer treatment.*

(Breast cancer, metastatic, female, age 70, unvaccinated).

*I am normally pro-vaccination but given my current diagnosis (terminal), I believe getting the vaccination could shorten my lifespan.*

(Gastrointestinal cancer, metastatic, female, age 61, unvaccinated).

#### 3.2.3. THEME 3. Uncertain Benefits of COVID-19 Vaccine Including Concerns about Vaccine Effectiveness

The COVID-19 vaccine was often described by participants as an “experimental injection” and for those with an altered immune system due to their chronic diseases, they felt particularly vulnerable. Many participants expressed their uncertainty regarding the benefits of the COVID-19 vaccines for them. In particular, the effectiveness of the vaccines for people with MS and cancer as some of the treatments are designed to or inadvertently reduce immune system activity.

*I am currently on Gilenya [Fingolimod]. Research says effectiveness is only 3.7%. This is why I am not getting vaccinated.*

(MS, relapsing-remitting, female, age 39, unvaccinated).

*I am worried that the efficacy [of the COVID-19 vaccine] will be lowered while undergoing treatment, especially chemotherapy.*

(Gastrointestinal cancer, localised, male, age 37, unvaccinated).

#### 3.2.4. THEME 4. Overwhelming Information Overload Disempowering Patients

Many participants reported feeling confused and overwhelmed by the information overload and frequent updates related to COVID-19 and vaccines. This was attributed to the numerous information sources including mass media, health care professionals, family, and social groups, which cause confusion leading to disempowerment for some people with informed decision making.

*I am very confused with which vaccine would be best for me. I am concerned about both the Pfizer AND the AstraZeneca [vaccines] and I am wondering whether I should wait for the Moderna vaccination. It is all SO confusing!!!!!*

(Lung cancer, localised, female, age 63, unvaccinated).

*Feeling unsure with all the negative commentary from some experts, [I] find it hard to trust and who to believe.*

(Type 2 diabetes, male, age 59, unvaccinated).

*I am concerned by the levels of misinformation that are circulating, perpetuated in large part by a media more interested in ratings and ‘clickbait’ than science.*

(Type 2 diabetes, male, age 66, vaccinated).

*I’m very confused, there are too many options and conspiracies that are tainting the facts, I just don’t know who to believe. I might even die before I know what’s real. It doesn’t matter if it’s COVID-19 or cancer. Dead is dead.*

(Genitourinary cancer, metastatic, male, age 59, unvaccinated).

Confusion was compounded by concerns related to the disease or treatments and anecdotal stories related to medical complications following vaccination.

*Because of all the medications I take, I’m worried that taking the vaccine will endanger my life. An American friend of mine has had the Moderna vaccines...since the 2nd dose she has been in and out of hospital and has had constant fever.*

(Type 2 diabetes, female, age 56, unvaccinated).

## 4. Discussion

This free-text qualitative analysis from a large survey of Australian adults with a significant underlying medical condition expands the understanding of intersecting concerns related to the impact of COVID-19 and COVID-19 vaccinations for people who live with a severe or chronic disease. It provides unique insights given there is currently a dearth of qualitative research undertaken to understand the nuances of COVID-19 vaccine hesitancy in medically vulnerable samples. It applies this to the HBM to maximize the utility of application in future policy and intervention development. It provides insight into the compounded complexity with the decision to accept COVID-19 vaccination in the context of disease- and treatment-related considerations.

Our in-depth analysis identified four dominant themes related to COVID-19 vaccination among respondents with an underlying disease: (1) having a chronic disease heightened perceived susceptibility to, and perceived severity of COVID-19; (2) perceived impact of vaccination on chronic disease management and disease-related safety; (3) uncertain benefits of COVID-19 vaccine including concerns about vaccine effectiveness; and (4) overwhelming information overload disempowering patients.

This study is the first to use the HBM as a theoretical framework to assess COVID-19 vaccine hesitancy behaviour across people with three different chronic diseases. It expands on previous studies that have applied the model to individual diseases, including diabetes and cancer [30,31]. Using this framework, we have established that a person’s underlying disease and treatment is an important consideration in vaccine acceptance/hesitancy and that concerns related to the vulnerability to COVID-19 infection are weighted with concerns about how the vaccination will impact a person’s disease. This is overlaid on COVID-19 vaccine concerns and conspiracies documented to be experienced by the general public more broadly [25].

Whilst vaccine hesitancy has been qualitatively explored within prioritised healthcare workers and patients with comorbidities [20,32], our study is unique in that it invited open-ended comments from participants of a large national study enabling us to undertake qualitative analysis on a very large sample. This allowed a large cross-section of views to be analysed and correlated with clinical status across three predefined high-risk disease types. Our research enhances the understanding of the decision-making implications an underlying health condition may have when weighing the costs and benefits of accepting a vaccine in the context of a person’s disease or treatment, adding to information previously identified using fixed responses within surveys [17,18,24,33,34].

### 4.1. Heightened Susceptibility and Severity to COVID-19

Heightened disease-related susceptibility to COVID-19 acquisition and severity was of particular concern for people with cancer and MS. Respondents with these diseases expressed concerns over impaired personal immunity from their underlying disease and/or treatment. Indeed, it is a commonality between these two diseases that immune response is a feature of the disease and may be impaired by some treatments [35,36,37,38]. It is of note that people with diabetes were less likely to state concerns about vulnerability to COVID-19 given that research early in the pandemic highlighted the vulnerability of this group [39]. While people with diabetes were appropriately prioritised in the vaccine rollout (Figure 1) [40], this suggests an issue about communicating COVID-19 risk to this group.

### 4.2. Disruption to Chronic Disease Management

Concerns about disruption to chronic disease management was a major theme. This included participants not wanting the vaccination while undergoing treatment, the impact on disease progression if the person needed to temporarily halt treatment to undergo vaccination, and the unknown long-term safety implication of vaccination on their chronic disease. Comments analysed within this theme demonstrated that even those who had accepted the vaccine grappled with difficulties reconciling perceived COVID-19 susceptibility with the risks of potential disruption of their chronic disease course or management. This was particularly evident for participants with cancer, where timing of COVID-19 vaccination is considered alongside treatment stage [41].

Similarly for MS, as certain immune-based therapies work by ameliorating the immune response and some reduce serologic response to COVID-19 vaccines [38], people reported the burden of weighing critical choices between prolonged isolation versus stopping their immunosuppression therapy to mount an immune response to the vaccine. The fear associated with reduced efficacy of either the vaccine or current disease-modifying therapy has been previously shown in a study of 1479 people with MS in Iran [42]. Participants across all diseases, but particularly the MS cohort, expressed concerns, such as “rocking the boat”, whilst their disease is stable. Concerns around vaccinations activating disease in MS are not new and a series of case reports propose potential disease activation in response to COVID-19 vaccines [43,44]. This highlights a need for research specific to the impact of vaccination on disease to confirm or debunk concerns, as well as up-to-date accurate advice from healthcare providers to promote shared decision making and reduce health-related anxiety. While there is evidence of immune system disruption in people with diabetes [45], it is less of a feature of the disease and did not appear to be a consideration for this cohort. Rather, people with diabetes expressed concerns about limited research into the long-term side effects of the vaccine on diabetes and those who assessed themselves as having poor glycaemic control (27% type 1 diabetes and 33% type 2 diabetes) were particularly concerned. This aligns with an Italian study that found poorer diabetes control was associated with vaccine hesitancy, particularly in people with type 1 diabetes, and poorer health more broadly was associated with vaccine hesitancy in people with type 2 diabetes [46].

### 4.3. Vaccine Safety and Efficacy

Similar to concerns about the rapidity of development of the vaccines seen in the general community and other chronic disease populations, our data reinforced safety and efficacy concerns balanced with COVID-19 disease and complication prevention and coupled with intersecting decision factors terms of the long-term impact on their disease [13,47,48].

Interestingly some participants with metastatic cancer raised concerns that the vaccination might not be safe given the impact of cancer and treatment on their immune system and others questioned the unknown benefits of vaccination given their reduced life expectancy. These concerns are likely compounded by evidence of a reduced vaccine proficiency for people treated with some cancer drugs [49]. People with cancer were excluded from initial COVID-19 vaccine safety and efficacy trials resulting in an initial evidence gap [49]. Longitudinal studies are required to document and generate data on the long-term safety and efficacy of COVID-19 vaccines in the context of chronic diseases to provide confidence in the acceptance of ongoing boosters. Given the fast-evolving nature of the pandemic, it remains critical that accurate and up-to-date disease specific information is distributed to relevant groups as it becomes available.

### 4.4. Information Overload and Inconsistency

The overwhelming information from competing sources, including mass media, healthcare professionals, family, and social groups confused and disempowered participants. This highlights the importance of targeting people with severe and chronic conditions who are particularly vulnerable to harms from misinformation. A previous study found that individuals educated about the vaccine by healthcare specialists were more likely to receive a vaccine, whilst misinformation spread by social media outlets predicted vaccine hesitancy [11]. It is well established that patients-consumers consider healthcare professionals a preferred and trusted source of information [50,51]. This highlights the need to appropriately resource healthcare providers to maximise accurate information distribution and reduce vaccine hesitancy.

Despite relatively high rates of COVID-19 vaccination, there were unexpectedly similar uptake rates between our prioritised at-risk sample and the general public [4]. Our quantitative analysis showed that 77.3% of unvaccinated participants were likely or unsure whether they would accept vaccination [4]. While multiple factors likely contribute to why these participants chose to delay rather than refuse vaccination, some participant comments highlighted a preference to wait for disease-specific evidence about the effects of vaccination. As evidence emerges it is important that targeted information is distributed to people with diseases so that COVID-19 vaccination rates are maximised.

Despite heterogeneity within and across the three diseases we studied in terms of disease duration and severity, there were many similarities in concerns over the vaccine’s potential or unknown interactions with their current treatment or disease status. The concept of ‘competing risks’ was particularly evident with relation to treatment disruption and disease progression. These intersectional decision-making factors are important to recognise in the context of COVID-19 vaccination, so that clinicians can address concerns at an individual level and public health authorities can target education to both patients and health care providers specific to various conditions. Our findings support the context-specific nature of vaccine hesitancy [9] that must be addressed to achieve COVID-19 vaccination behavioural change for people with chronic diseases. It is important that healthcare providers are appropriately resourced to address concerns of people with underlying health conditions, especially as future variants emerge and further research emerges to address perceived risks and information gaps specific for disease subgroups.

### 4.5. Implications for Future Research and Practice

Application of the qualitative findings from the current study to the HBM enables development of future policy and interventions that is evidence-based but applied to a theoretical framework. In particular, this emphasizes the interacting decision-making factors of people with serious medical conditions, that include implications due to their disease, as well as broader concerns and conspiracies.

This study highlights the need for clear information about disease management, when and how to manage disruptions to treatment, if required. This includes information that is targeted towards medical practitioners and can be individualized to the patient’s context.

A proactive approach in the development and distribution of accurate and targeted information for healthcare professionals and patients will assist with providing patient confidence in vaccination timing. Information should be delivered to patients by a trusted medical professional and supported by written literature to reduce inconsistency, recall accuracy, and information overload.

Notwithstanding that the COVID-19 pandemic was a rapidly evolving situation, consideration should be given to the practicalities of including participants from medically vulnerable populations at the outset in future studies to improve confidence in people from these populations when asked to accept vaccination.

### 4.6. Strengths and Limitations

During the data collection period, Australia had relatively low rates of community transmission and mortality due to COVID-19 compared to the rest of world, which should be considered with relation to generalisability of our results [52]. However, our qualitative themes are developed from a large sample which should provide confidence in their robustness. The multicultural makeup of the Australian population and the similarities in COVID-19 vaccination prioritization to vulnerable medical populations support the international translation of study findings. As our survey was only available in English, it is less likely to capture the opinions of linguistically diverse populations who we know have unique needs [53].

Despite these limitations, our in-depth analysis using a large sample adds to the accumulating literature documenting the needs of individuals with chronic diseases for access to information regarding the efficacy and safety of COVID-19 vaccines in the context of potential impact on their comorbid disease(s) [20,32,54]. Our study method of analysing undirected survey comments enabled unbiased and unguided identification of concerns related to vaccine hesitancy in people with chronic disease.

While the study invitation was distributed to all participants attending participating healthcare services within a specific timeframe to limit recruitment bias, it is possible that people who were interested in COVID-19 vaccination were more likely to respond. This analysis was limited to the views of the 34.3% of respondents that provided free-text comments when completing a larger survey. Comparative analysis between commenters and non-commenters highlighted they were broadly similar, although unvaccinated participants and females were more likely to comment, while participants with diabetes were less likely to comment compared to those with cancer or MS. It is, therefore, possible that these themes do not represent the broader survey respondents.

## 5. Conclusions

This qualitative analysis of free-text responses from Australians with cancer, diabetes, and MS highlights that many remain concerned about the complex interactions and uncertainties regarding vaccines in the context of their chronic disease. The four main themes identified were similar across diseases and included heightened concerns about susceptibility and severity to COVID-19, disruption to chronic disease management, vaccine safety and efficacy in the context of their disease, and information overload. Understanding these concerns will allow both individual health care providers and public policy to target information to those most vulnerable to poor outcomes from COVID-19 infection.

## Figures and Tables

**Table 1:** Characteristics of participants who provided free-text comments by underlying diagnosis.

Characteristic	All n = 1604 n, (%)	Cancer n = 1248 n, (%)	Diabetes n = 244 n, (%)	MS n = 112 n, (%)
**Gender ^a^**				
Male	658 (41.0)	511 (40.9)	120 (49.2)	27 (24.1)
Female	937 (58.4)	730 (58.5)	124 (50.8)	83 (74.1)
**Age (years)**				
Median (Range)	62.0 (19.0–88.0)	64.0 (20.0–88.0)	58.0 (19.0–87.0)	47.0 (19.0–77.0)
18–29	34 (2.1)	9 (0.7)	14 (5.7)	11 (9.8)
30–49	272 (17.0)	166 (13.3)	54 (22.1)	52 (46.4)
50–69	876 (54.6)	687 (55.0)	145 (59.4)	44 (39.3)
≥70	422 (26.3)	386 (30.9)	31 (12.7)	5 (4.5)
**Highest level of education ^b^**				
No formal/primary school	40 (2.5)	27 (2.2)	10 (4.1)	3 (2.7)
Secondary school	450 (28.1)	340 (27.2)	79 (32.4)	31 (27.7)
Vocational/Trade	416 (25.9)	320 (25.6)	69 (28.3)	27 (24.1)
University	693 (43.2)	559 (44.8)	84 (34.4)	50 (44.6)
**Annual household income (AUD)**				
<50 K	530 (33.0)	405 (32.5)	99 (40.6)	26 (23.2)
50–100 K	411 (25.6)	310 (24.8)	68 (27.9)	33 (29.5)
100 K–150 K	213 (13.3)	174 (13.9)	23 (9.4)	16 (14.3)
>150 K	188 (11.7)	153 (12.3)	20 (8.2)	15 (13.4)
Prefer not to say	262 (16.3)	206 (16.5)	34 (13.9)	22 (19.6)
**Aboriginal/Torres Strait Islander ^c^**				
Yes	23 (1.4)	18 (1.4)	5 (2.0)	0 (0.0)
No	1558 (97.1)	1212 (97.1)	234 (95.9)	112 (100.0)
**English as dominant language**				
Yes	1453 (90.6)	1150 (92.1)	203 (83.2)	100 (89.3)
No	151 (9.4)	98 (7.9)	41 (16.8)	12 (10.7)
**Location**				
Metropolitan	1072 (66.8)	808 (64.7)	171 (70.1)	93 (83.0)
Regional	532 (33.2)	440 (35.3)	73 (29.9)	19 (17.0)
**Location (state)**				
Victoria	669 (41.7)	433 (34.7)	143 (58.6)	93 (83.0)
New South Wales	592 (36.9)	553 (44.3)	39 (16.0)	0 (0.0)
Border (Victoria/New South Wales)	12 (0.7)	12 (1.0)	0 (0.0)	0 (0.0)
Queensland	247 (15.4)	166 (13.3)	62 (25.4)	19 (17.0)
Tasmania	84 (5.2)	84 (6.7)	0 (0.0)	0 (0.0)
**Vaccination status**				
1 dose	321 (20.0)	270 (21.6)	39 (16.0)	12 (10.7)
2 doses	841 (52.4)	636 (51.0)	132 (54.1)	73 (65.2)
No doses	442 (27.6)	342 (27.4)	73 (29.9)	27 (24.1)
**Vaccination intent ^d^**				
Likely	1330 (82.9)	1056 (84.6)	183 (75.0)	91 (81.3)
Unsure	141 (8.8)	102 (8.2)	28 (11.5)	11 (9.8)
Unlikely	133 (8.3)	90 (7.2)	33 (13.5)	10 (8.9)

^a^ There was ‘Non-binary/prefer not to say’ (n = 9, 0.6%). ^b^ There was also ‘Other’ (n = 5, 0.3%). ^c^ There was also ‘Prefer not to say’ (n = 23, 1.4%). ^d^ Vaccination intent were categorised as likely, have/definitely/probably; unsure, may or may not/possibly/don’t know; unlikely, probably not/definitely not. Abbreviations: MS, Multiple sclerosis; AUD, Australian Dollars; K, 1000.

## Data Availability

The data presented in this study are available on reasonable request from the corresponding author.

## Supplementary Materials

The following supporting information can be downloaded at: https://www.mdpi.com/article/10.3390/vaccines11020239/s1, List of CANVACCS, DIABVACCS, and MSVACCS investigators; Table S1: Clinical characteristics of participants who provided free-text comments, by underlying diagnosis; Table S2: Chi-squared analyses for comparison of characteristics in participants with cancer, by commenter status; Table S3: Chi-squared analyses for comparison of characteristics in participants with diabetes, by commenter status; Table S4: Chi-squared analyses for comparison of characteristics in participants with multiple sclerosis, by commenter status.
