# Supplementary material for: Factors Influencing COVID-19 Vaccine Hesitancy among Patients with Serious Chronic Illnesses during the Initial Australian Vaccine Rollout: A Multi-Centre Qualitative Analysis Using the Health Belief Model
Source: Vaccines (Basel). 2023 Jan 20;11(2):239. doi: 10.3390/vaccines11020239 (PMC9963130; doi:10.3390/vaccines11020239)
Supplement: Supplementary file 1 [file vaccines-11-00239-s001.zip › vaccines-2148200-supplementary.pdf]

Supplementary materials

These supplementary materials have been provided by the authors to give readers additional information about their work.

List of CANVACCS, DIABVACCS, and MSVACCS investigators

Monash Health, Victoria

CANVACCS: Dr. Veronica Lopez Aedo, Dr. Elizabeth Ahern, Dr. Muhammad Alamgeer, Dr. Nathan Bain, Dr. Amy Body, A/Prof. Peter Briggs, Dr. Daphne Day, Dr. Sophia Frentzas, Dr. Lisa Grech, A/Prof. Marion Harris, Dr. Gwo-Yaw Ho, Dr. Caroline Lum, Dr. Vi Luong, Dr. Amelia McCartney, Dr. Cameron McLaren, Dr. Mike Nguyen, Prof. Stephen Opat, A/Prof. David Pook, Prof. Eva Segelov, A/Prof. Andrew Strickland, Dr. Avraham Travers, Dr. Kate Webber, Dr. Michelle White, Dr. Walid Zwieky.

DIABVACCS: Prof. Barbora de Courten, A/Prof. Jennifer Wong.

MSVACCS: Ms Michelle Allan, A/Prof. Ernest Butler.

Bendigo Health, Victoria

CANVACCS: Dr. Sam Harris.

DIABVACCS: Dr. Frank Gao, Dr. Amy Harding, A/Prof. Mark Savage.

Latrobe Regional Hospital, Victoria

CANVACCS: Dr. Hieu Chau.

Sunshine Coast Hospital and Health Service, Queensland

CANVACCS: A/Prof. Bryan Chan.

DIABVACCS: Dr. Brett Sillars.

MSVACCS: Dr. Joshua Barton, Dr. Antony Winkel.

Icon Cancer Centre Hobart, Tasmania

CANVACCS: A/Prof. Louise Nott.

Central Coast Haematology, New South Wales

CANVACCS: Dr. Richard Blennerhassett, Dr. Cecily Forsyth, Ms. Jacqueline Jagger.

St Vincent's Hospital Sydney, New South Wales

CANVACCS: A/Prof. Nada Hamad.

Dr David Hoffman, New South Wales

DIABVACCS: Dr. David Hoffman.

Border Medical Oncology Research Unit, New South Wales

CANVACCS: A/Prof. Craig Underhill.

Table S1. Clinical characteristics of participants who provided free-text comments, by underlying diagnosis (n = 1604).

Characteristic	Cancer n = 1248 n, (%)	Diabetes n = 244 n, (%)	MS n = 112 n, (%)
**Cancer type**			
Breast	355 (28.4)		
Gastrointestinal	143 (11.5)		
Blood	354 (28.4)		
Genitourinary	147 (11.8)		
Lung	87 (7.0)		
Gynaecological	47 (3.8)		
Skin	56 (4.5)		
Head and neck	32 (2.6)		
Brain	17 (1.4)		
Other	10 (0.8)		
**Cancer stage[a]**			
Localised	524 (58.7)		
Metastatic	333 (37.3)		
Don't Know/Other	36 (4.0)		
**Time since diagnosis**			
<6 months	149 (11.9)		
6 – 24 months	390 (31.3)		
2 – 5 years	386 (30.9)		
>5 years	323 (25.9)		
**Currently on anti-cancer treatment**			
Yes	636 (51.0)		
No	612 (49.0)		
**Diabetes type**			
Type 1		90 (36.9)	
Type 2		145 (59.4)	
Other/Don't know		9 (3.7)	
**Time since diabetes diagnosis**			
<1 year		9 (3.7)	
1 – 5 years		30 (12.3)	
5.1 – 10 years		45 (18.4)	
>10 years		160 (65.6)	
**Most recent HbA1c within the past year**			
<7%		40 (16.5)	
7% – 8.5%		110 (45.3)	
8.6 – 10%		48 (19.8)	
>10%		15 (6.2)	
Don't know		30 (12.3)	
**Current diabetes treatment**			
Insulin		90 (36.9)	
Tablets		40 (16.4)	
Injectables (not insulin)		4 (1.6)	
Diet only		5 (2.0)	
Combination of treatments/Other		105 (43.0)	
**Management of diabetes in the past month:**			
Excellent		25 (10.3)	
Very good		62 (25.5)	
Good		80 (32.9)	
Fair		54 (22.2)	
Poor		22 (9.1)	
**Diabetes affect daily activities in last 4 weeks**			
All of the time		20 (8.2)	

Most of the time	29 (11.9)	
Some of the time	66 (27.2)	
Not very often	64 (26.3)	
Not at all	64 (26.3)	
**Multiple sclerosis (MS) type**		
Relapsing-remitting MS (RRMS)		87 (77.7)
Primary progressive MS (PPMS)		8 (7.1)
Secondary progressive MS (SPMS)		10 (8.9)
Other/Don’t know		7 (6.3)
**Time since MS diagnosis**		
<1 year		3 (2.7)
1 – 5 years		37 (33.0)
5.1 – 10 years		23 (20.5)
>10 years		49 (43.8)
**Current MS treatment**		
Tablets		37 (33.0)
Injectables		11 (9.8)
Intravenous		42 (37.5)
No specific treatment/Other		22 (19.6)
**MS control over past 6 months**		
Yes		88 (78.6)
No		13 (11.6)
Don’t know		11 (9.8)
**No. of times missed disease modifying therapies in the past month**		
All of the time		4 (3.6)
Most of the time		0 (0.0)
Some of the time		3 (2.7)
Occasionally		13 (11.8)
Never		90 (81.8)
**MS affect daily activities in last 4 weeks**		
All of the time		14 (12.5)
Most of the time		12 (10.7)
Some of the time		44 (39.3)
Not very often		20 (17.9)
Not at all		22 (19.6)

[a] There was also ‘Not applicable/blood cancer’ (n = 355). Abbreviations: MS, multiple sclerosis; HbA1c, Haemoglobin A1C.

Table S2. Chi-squared analyses for comparison of characteristics in participants with cancer, by commenter status (n = 3560).

	Did not comment (n = 2312) n, (%)	Commented (n = 1248) n, (%)	X^2 p-value[a]	Cramer’s V
**Gender**			0.002	0.05*
Male	1075 (67.8)	511 (32.2)		
Adj. residuals	3.2	-3.2		
Female	1227 (62.7)	730 (37.3)		
Adj. residuals	-3.2	3.2		
**Age**			0.06	0.04
18 – 49	335 (65.7)	175 (34.3)		
Adj. residuals	0.4	-0.4		
50 – 69	1177 (63.1)	687 (36.9)		
Adj. residuals	-2.3	2.3		
≥70	796 (67.3)	386 (32.7)		
Adj. residuals	2.2	-2.2		

**Highest level of education**			<0.001	0.10
No formal / Primary school	63 (70.0)	27 (30.0)		
Adj. residuals	1.0	1.0		
Secondary school	827 (70.9)	340 (29.1)		
Adj. residuals	5.2	-5.2		
Vocational/Trade	576 (64.3)	320 (35.7)		
Adj. residuals	-0.4	0.4		
University	837 (60.0)	559 (40.0)		
Adj. residuals	-5.0	5.0		
**Annual household income (AUD)**			0.40	0.03
<50K	750 (64.9)	405 (35.1)		
Adj. residuals	0.0	0.0		
50-100K	544 (63.7)	310 (36.3)		
Adj. residuals	-0.9	0.9		
100K-150K	288 (62.3)	174 (37.7)		
Adj. residuals	-1.3	1.3		
>150K	311 (67.0)	153 (33.0)		
Adj. residuals	1.0	-1.0		
Prefer not to say	419 (67.0)	206 (33.0)		
Adj. residuals	1.2	-1.2		
**English as dominant language**			0.10	-0.03*
Yes	2090 (64.5)	1150 (35.5)		
No	221 (69.3)	98 (30.7)		
**Location**			0.028	0.04*
Metropolitan	1582 (66.2)	808 (33.8)		
Adj. residuals	2.2	-2.2		
Regional	730 (62.4)	440 (37.6)		
Adj. residuals	-2.2	2.2		
**Vaccination status[b]**			<0.001	0.16*
Yes	1978 (68.6)	906 (31.4)		
Adj. residuals	9.5	-9.5		
No	332 (49.3)	342 (50.7)		
Adj. residuals	-9.5	9.5		
**Vaccination intent[c]**			<0.001	0.15
Likely	2161 (67.2)	1056 (32.8)		
Adj. residuals	8.6	-8.6		
Unsure/Don't know	99 (49.3)	102 (50.7)		
Adj. residuals	-4.8	4.8		
Unlikely	51 (36.2)	90 (63.8)		
Adj. residuals	-7.3	7.3		
**Belief about likelihood of COVID-19 infection[d]**			0.001	0.06
Likely	994 (62.9)	587 (37.1)		
Adj. residuals	-2.3	2.3		
Unlikely	877 (68.9)	396 (31.1)		
Adj. residuals	3.7	-3.7		
Don't know	441 (62.5)	265 (37.5)		
Adj. residuals	-1.5	1.5		
**Cancer type**			0.001	0.09
Breast	631 (64.0)	355 (36.0)		
Adj. residuals	-0.7	0.7		
Gastrointestinal	350 (71.0)	143 (29.0)		
Adj. residuals	3.0	-3.0		
Blood	515 (59.3)	354 (40.7)		
Adj. residuals	-4.0	4.0		
Genitourinary	354 (70.7)	147 (29.3)		
Adj. residuals	2.9	-2.9		
Lung	161 (64.9)	87 (35.1)		

Adj. residuals	0.0	0.0		
Gynaecological	87 (64.9)	47 (35.1)		
Adj. residuals	0.0	0.0		
Skin	95 (62.9)	56 (37.1)		
Adj. residuals	-0.5	0.5		
Head and neck	67 (67.7)	32 (32.3)		
Adj. residuals	0.6	-0.6		
Brain	29 (63.0)	17 (37.0)		
Adj. residuals	-0.3	0.3		
Other	23 (69.7)	10 (30.3)		
Adj. residuals	0.6	-0.6		
**Cancer stage**			0.49	0.01*
Localised	1069 (67.1)	524 (32.9)		
Adj. residuals	0.7	-0.7		
Metastatic	638 (65.7)	333 (34.3)		
Adj. residuals	-0.7	0.7		
**Time since cancer diagnosis**			0.61	0.02
<6 months	287 (65.8)	149 (34.2)		
Adj. residuals	0.4	-0.4		
6-24 months	758 (66.0)	390 (34.0)		
Adj. residuals	0.9	-0.9		
2-5 years	710 (64.8)	386 (35.2)		
Adj. residuals	-0.1	0.1		
>5 years	557 (63.3)	323 (36.7)		
Adj. residuals	-1.2	1.2		
**Current anti-cancer treatment**				
Yes	1172 (64.8)	636 (35.2)	0.88	-0.003*
Adj. residuals	-0.2	0.2		
No	1140 (65.1)	612 (34.9)		
Adj. residuals	0.2	-0.2		

* Phi coefficient reported. Abbreviations: Adj. residuals, adjusted residuals; AUD, Australian Dollars; K, 1000. [a] Chi-square p-value. The following categories were not included in the chi-square analyses: Non-binary/prefer not to say (gender); other (highest level of education); don't know/other/not applicable (cancer stage). [b] Vaccination status was defined as receiving at least one COVID-19 vaccine dose. [c] Vaccination intent were categorised as: Likely, Have/definitely/probably; Unsure, May or may not/possibly/don't know; Unlikely, Probably not/definitely not. [d] Belief about likelihood of contracting COVID-19 within the next 12 months were categorised as: Likely, Definitely/probably/possibly; Unlikely, Probably not/Definitely Not; Don't know, Don't know.

Table S3. Chi-squared analyses for comparison of characteristics in participants with diabetes, by commenter status (n = 842).

	Did not comment (n = 598) n, (%)	Commented (n = 244) n, (%)	X^2 p-value[a]	Cramer's V
**Gender**			0.046	0.07*
Male	337 (73.7)	120 (26.3)		
Adj. residuals	2.1	-2.1		
Female	254 (67.2)	124 (32.8)		
Adj. residuals	-2.1	2.1		
**Age**			0.09	0.08
18 – 49	183 (72.9)	68 (27.1)		
Adj. residuals	0.8	-0.8		
50 – 69	310 (68.1)	145 (31.9)		
Adj. residuals	-2.0	2.0		
≥70	105 (77.2)	31 (22.8)		

Adj. residuals	1.7	-1.7		
**Highest level of education**			0.16	0.08
No formal/Primary school	27 (73.0)	10 (27.0)		
Adj. residuals	0.2	-0.2		
Secondary school	240 (75.2)	79 (24.8)		
Adj. residuals	2.0	-2.0		
Vocational/Trade	162 (70.1)	69 (29.9)		
Adj. residuals	-0.4	0.4		
University	169 (66.8)	84 (33.2)		
Adj. residuals	-1.8	1.8		
**Annual household income (AUD)**			0.08	0.10
<50K	224 (69.3)	99 (30.7)		
Adj. residuals	-0.8	0.8		
50-100K	149 (68.7)	68 (31.3)		
Adj. residuals	-0.9	0.9		
100K-150K	69 (75.0)	23 (25.0)		
Adj. residuals	0.9	-0.9		
>150K	32 (61.5)	20 (38.5)		
Adj. residuals	-1.6	1.6		
Prefer not to say	124 (78.5)	34 (21.5)		
Adj. residuals	2.3	-2.3		
**English as dominant language**			0.23	-0.05*
Yes	474 (70.0)	203 (30.0)		
Adj. residuals	-1.3	1.3		
No	124 (75.2)	41 (24.8)		
Adj. residuals	1.3	-1.3		
**Location**			0.52	0.03*
Metropolitan	434 (71.7)	171 (28.3)		
Adj. residuals	0.7	-0.7		
Regional	164 (69.2)	73 (30.8)		
Adj. residuals	-0.7	0.7		
**Vaccination status[b]**			<0.001	0.21*
Yes	525 (75.4)	171 (24.6)		
Adj. residuals	6.2	-6.2		
No	73 (50.0)	73 (50.0)		
Adj. residuals	-6.2	6.2		
**Vaccination intent[c]**			<0.001	0.27
Likely	559 (75.3)	183 (24.7)		
Adj. residuals	7.5	-7.5		
Unsure/Don’t know	24 (46.2)	28 (53.8)		
Adj. residuals	-4.1	4.1		
Unlikely	15 (31.3)	33 (68.6)		
Adj. residuals	-6.3	6.3		
**Belief about likelihood of COVID-19 infection[d]**			0.55	0.04
Likely	272 (69.6)	119 (30.4)		
Adj. residuals	-0.9	0.9		
Unlikely	187 (71.1)	76 (28.9)		
Adj. residuals	0.0	0.0		
Don’t know	139 (73.9)	49 (26.1)		
Adj. residuals	1.0	-1.0		
**Diabetes type**			0.005	-0.10*
Type 1	162 (64.3)	90 (35.7)		
Adj. residuals	-2.8	2.8		
Type 2	412 (74.0)	145 (26.0)		
Adj. residuals	2.8	-2.8		
**Time since diabetes diagnosis**			0.61	0.05
<1 year	28 (75.7)	9 (24.3)		

Adj. residuals	0.6	-0.6		
1 – 5 years	76 (71.7)	30 (28.3)		
Adj. residuals	0.2	-0.2		
5.1 – 10 years	129 (74.1)	45 (25.9)		
Adj. residuals	1.0	-1.0		
>10 years	365 (69.5)	160 (30.5)		
Adj. residuals	-1.2	1.2		
**Most recent Haemoglobin A1C (HbA1c) within the past year**			0.03	0.11
<7%	99 (71.2)	40 (28.8)		
Adj. residuals	0.1	-0.1		
7% to 8.5%	224 (67.1)	110 (32.9)		
Adj. residuals	-2.0	2.0		
8.6 to 10%	100 (67.6)	48 (32.4)		
Adj. residuals	-1.0	1.0		
>10%	52 (77.6)	15 (22.4)		
Adj. residuals	1.2	-1.2		
Don't know	120 (80.0)	30 (20.0)		
Adj. residuals	2.7	-2.7		
**Current diabetes treatment**				
Yes	587 (71.1)	239 (28.9)	0.79	0.01*
Adj. residuals	0.2	-0.2		
No	11 (68.8)	5 (31.3)		
Adj. residuals	-0.2	0.2		
**Management of diabetes in the past month:**			0.03	0.11
Excellent	62 (71.3)	25 (28.7)		
Adj. residuals	0.0	0.0		
Very good	175 (73.8)	62 (26.2)		
Adj. residuals	1.1	-1.1		
Good	222 (73.5)	80 (26.5)		
Adj. residuals	1.2	-1.2		
Fair	115 (68.0)	54 (32.0)		
Adj. residuals	-1.0	1.0		
Poor	24 (52.2)	22 (47.8)		
Adj. residuals	-2.9	2.9		
**Diabetes affect daily activities in last 4 weeks**			0.44	0.07
All of the time	32 (61.5)	20 (38.5)		
Adj. residuals	-1.6	1.6		
Most of the time	63 (68.5)	29 (31.5)		
Adj. residuals	-0.6	0.6		
Some of the time	156 (70.3)	66 (29.7)		
Adj. residuals	-0.3	0.3		
Not very often	165 (72.1)	64 (27.9)		
Adj. residuals	0.4	-0.4		
Not at all	182 (74.0)	64 (26.0)		
Adj. residuals	1.2	-1.2		

* Phi coefficient reported. Abbreviations: Adj. residuals, adjusted residuals; AUD, Australian Dollars; K, 1000; HbA1c, Haemoglobin A1C. [a] Chi-square p-value. The following categories were not included in the analyses: Non-binary/prefer not to say (gender); other (highest level of education); don't know/other (diabetes type). [b] Vaccination status was defined as receiving at least one COVID-19 vaccine dose. [c] Vaccination intent were categorised as: Likely, Have/definitely/probably; Unsure, May or may not/possibly/don't know; Unlikely, Probably not/definitely not. [d] Belief about likelihood of contracting COVID-19 within the next 12 months were categorised as: Likely, Definitely/probably/possibly; Unlikely, Probably not/Definitely Not; Don't know, Don't know.

Table S4. Chi-squared analyses for comparison of characteristics in participants with multiple sclerosis, by commenter status (n = 281).

	Did not comment (n = 169) n, (%)	Commented (n = 112) n, (%)	X^2 p-value[a]	Cramer's V
**Gender**			0.71	-0.02*
Male	38 (58.5)	27 (41.5)		
Adj. residuals	-0.4	0.4		
Female	130 (61.0)	83 (39.0)		
Adj. residuals	0.4	-0.4		
**Age**			0.60	0.06
18 – 39	47 (56.6)	36 (43.4)		
Adj. residuals	-0.8	0.8		
40 – 59	83 (60.1)	55 (39.9)		
Adj. residuals	0.0	0.0		
≥60	39 (65.0)	21 (35.0)		
Adj. residuals	0.9	-0.9		
**Highest level of education**			0.56	0.06
No formal/primary school/secondary school	56 (61.5)	35 (38.5)		
Adj. residuals	0.3	-0.3		
Vocational/Trade	48 (64.0)	27 (36.0)		
Adj. residuals	0.8	-0.8		
University	65 (56.5)	50 (43.5)		
Adj. residuals	-1.0	1.0		
**Annual household income (AUD)**			0.90	0.06
<50K	46 (63.9)	26 (36.1)		
Adj. residuals	0.8	-0.8		
50 – 100K	43 (56.6)	33 (43.4)		
Adj. residuals	-0.7	0.7		
100K – 150K	25 (61.0)	16 (39.0)		
Adj. residuals	0.1	-0.1		
>150K	25 (62.5)	15 (37.5)		
Adj. residuals	0.3	-0.3		
Prefer not to say	30 (57.7)	22 (42.3)		
Adj. residuals	-0.4	0.4		
**English as dominant language**			0.40	0.06*
Yes	157 (61.1)	100 (38.9)		
Adj. residuals	1.1	-1.1		
No	12 (50.0)	12 (50.0)		
Adj. residuals	-1.1	1.1		
**Location**			0.44	0.05*
Metropolitan	146 (61.1)	93 (83.9)		
Adj. residuals	0.8	-0.8		
Regional	23 (54.8)	19 (45.2)		
Adj. residuals	-0.8	0.8		
**Vaccination status[b]**			0.02	0.15*
Yes	148 (63.5)	85 (36.5)		
Adj. residuals	2.5	-2.5		
No	21 (43.8)	27 (56.3)		
Adj. residuals	-2.5	2.5		
**Vaccination intent[c]**			0.006	0.19
Likely	158 (63.5)	91 (36.5)		
Adj. residuals	3.2	-3.2		
Unsure/Don't know	7 (38.9)	11 (61.1)		
Adj. residuals	-1.9	1.9		
Unlikely	4 (28.6)	10 (71.4)		
Adj. residuals	-2.5	2.5		
**Belief about likelihood of COVID-19 infection[d]**			0.85	0.03
Likely	85 (59.4)	58 (40.6)		
Adj. residuals	-0.2	0.2		

Unlikely	54 (59.3)	37 (40.7)		
Adj. residuals	-0.2	0.2		
Don't know	30 (63.8)	17 (36.2)		
Adj. residuals	0.6	-0.6		
**Multiple sclerosis (MS) type**			0.35	0.09
Relapsing-remitting MS	116 (57.1)	87 (42.9)		
Adj. residuals	-1.4	1.4		
Primary progressive MS	18 (69.2)	8 (30.8)		
Adj. residuals	1.1	-1.1		
Secondary progressive MS	20 (66.7)	10 (33.3)		
Adj. residuals	0.9	-0.9		
**Time since MS diagnosis[e]**			0.39	0.08
<5 years	48 (54.5)	40 (45.5)		
Adj. residuals	-1.3	1.3		
5.1 – 10 years	35 (60.3)	23 (39.7)		
Adj. residuals	0.0	0.0		
> 10 years	86 (63.7)	49 (36.3)		
Adj. residuals	1.2	-1.2		
**Current MS treatment**			0.30	0.10
Tablets	69 (65.1)	37 (34.9)		
Adj. residuals	1.5	-1.5		
Injectables	15 (57.7)	11 (42.3)		
Adj. residuals	-0.2	0.2		
Intravenous	50 (54.3)	42 (45.7)		
Adj. residuals	-1.4	1.4		
**MS control over past 6 months**			0.65	0.06
Yes	138 (61.1)	88 (38.9)		
Adj. residuals	0.6	-0.6		
No	14 (51.9)	13 (48.1)		
Adj. residuals	-0.9	0.9		
Don't know	17 (60.7)	11 (39.3)		
Adj. residuals	0.1	-0.1		
**MS affect daily activities in last 4 weeks**			0.11	0.16
All of the time	30 (68.2)	14 (31.8)		
Adj. residuals	1.2	-1.2		
Most of the time	23 (65.7)	12 (34.3)		
Adj. residuals	0.7	-0.7		
Some of the time	41 (48.2)	44 (51.8)		
Adj. residuals	-2.7	2.7		
Not very often	33 (62.3)	20 (37.7)		
Adj. residuals	0.4	-0.4		
Not at all	42 (65.6)	22 (34.4)		
Adj. residuals	1.0	-1.0		

* Phi coefficient reported. Abbreviations: Adj. residuals, adjusted residuals; AUD, Australian Dollars; K, 1000; MS, multiple sclerosis. [a] Chi-square p-value. The variable 'No. of times missed disease-modifying therapies in the last month' was not analysed due to low frequencies in most of its categories. The following categories were not included in the analyses: Non-binary/prefer not to say (gender); other (highest level of education); don't know/other (MS type). [b] Vaccination status was defined as receiving at least one COVID-19 vaccine dose. [c] Vaccination intent were categorised as: Likely, Have/definitely/probably; Unsure, May or may not/possibly/don't know; Unlikely, Probably not/definitely not. [d] Belief about likelihood of contracting COVID-19 within the next 12 months were categorised as: Likely, Definitely/probably/possibly; Unlikely, Probably not/Definitely Not; Don't know, Don't know.[e] The '<1 year' and '1 – 5 years' categories were combined for analysis.
